# Methodological considerations for analyzing enriched enrolment randomized withdrawal trials

**DOI:** 10.1016/j.clinsp.2026.101131

**Published:** 2026-09-09

**Authors:** Philippe Lehert

**Affiliations:** a School of Management, UC Louvain University, Belgium; b Faculty of Medicine, The University of Melbourne, Australia

**Keywords:** Enriched Enrolment randomized withdrawal (EERW), Randomized withdrawal Trial RWT, ANCOCA analysis of covariance, Statistical power

## Abstract

**Background:**

The Enriched Enrolment Randomized Withdrawal (EERW) design has been widely employed under various names across multiple disease areas. The methodology underlying these trials remains subject to ongoing discussion. A review of the literature identified key criticisms associated with the EERW design, notably the Open-Label Effect (OLE) resulting from the initial Open-Label (OL) phase and the Selection Effect (SLE) arising from selecting responders at the conclusion of the OL period. The aim of this study was to quantify these effects using historical data and to propose a straightforward approach to mitigate their impact.

**Methods:**

Based on proposals to estimate SLE and OLE effects, we conducted a retrospective analysis on a sample of 42 EERWs studies in various disorders and pathologies.

**Results:**

The OLE effect was confirmed with a highly significant additional increase of the main final endpoint (Standardized effect β_std_ = 0.567, p < 0.001). Likewise, the SLE was observed with a smaller but significant effect (β_std_ = 0.263, p = 0.028). Adding OLE and SLE effects in the covariance model assessing the significance of the studied treatment effect provided a highly significant increase of the determination coefficient ΔR^2^ = 0.380 (p < 0.001) reducing the residual variability of the model. The treatment effect was not significantly affected by these effects (p > 0.150).

**Conclusion:**

While EERW designs are widely utilized, the impact of the Open-Label (OL) phase on statistical outcomes remains under-addressed. This study quantifies two distinct and specific OLE and SLE effects increasing the residual variability and reducing the precision of the studied treatment estimate. By incorporating these effects as adjustments within the primary inferential model, researchers can mitigate extraneous noise and improve the precision and robustness of EERW trial results.

## Highlights

• EERW designs are widely utilized, but their methodology remains a subject of ongoing debate.
• This study investigates the effects arising from the Open-Label (OL) phase and the subsequent selection of patients (OLE and SLE effects respectively).
• We attempted these effects demonstrating that while they may not directly bias the treatment effect estimate.
• However, they significantly increase residual variability.
• By incorporating these two effects as adjustments within the inferential model, this extraneous variability can be mitigated, leading to more precise and robust statistical outcomes.

## Background

Compared with classical Randomized Controlled Trials (RCT), an Enriched Enrolment Randomized Withdrawal trial (EERW), alias Randomized Withdrawal Trial (RWT) starts with an Open-Label (OL) Period in which patients receive the studied treatment, followed by a second phase starting by enrolment and randomization to the studied treatment versus a control group.

EERW trials were pioneered as early as 1975.^1^ with various appellations and used for the first time for regulatory purposes^2^ for a trial of nifedipine, subsequently approved by the US Food and Drug Administration [FDA] In 2012, the FDA officially endorsed EERW designs for regulatory approval, without providing specific guidelines.^3^ EERWs were later more often used in some disorders, such as chronic pain trials.^4^

The most common EERW design is the Complete Enriched Enrolment (CEE) model,^5^^,^^6^ where patients who meet pre-established response criteria for both efficacy and safety at the end of the Open-Label (OL) phase are enrolled.

Another approach, known as Partial Enriched Enrolment (PEE),^5^ involves enrolling both responders from the OL phase and a selection of nonresponses. A different proposed method is to use a Randomized Controlled Trial (RCT) to identify drug responders, who are then entered into another RCT comparing the study drug with placebo.^7^ Responders may also be classified as individuals who take the drug and report experiencing benefits.^8^ An alternative strategy is to discontinue treatment in patients who respond to the drug, then enroll only those whose condition worsens after stopping the study medication from the initial RCT.^4^

The advantage of EERWs can first be summarized from a statistical perspective: higher statistical power was claimed.^9^ no apparent bias on results of efficacy, but possible underestimation of the adverse effects.^10^ Clinical advantages include sustaining improvement in responsive patients,^11^^,^^12^ more efficient resource use and quicker drug development in some cases, and closer alignment with routine practice where only responders continue treatment.^13^

Among EERW options^.^^3^^,^^5^^,^^13^ in the context of pediatrics, the Complete EERW (CEE) maximizes the likelihood of observing a treatment effect during the randomized withdrawal phase, as the study population is enriched with individuals who are likely to benefit from the therapy.

A PEE can offer clearer information about the consistency of treatment effects at different initial response levels, helping identify which subgroups benefit most from long-term therapy. Facing these potential benefits, recent publications have provided criticism on EERWs. First, doubts were suspected concerning statistical power and EERW validity when studying a chronic disease without frequent episodic recovery.^14^ Similar criticisms arose in chronic pain, where treatments do not cure or significantly change the disease’s course.^15^

Severe lacks of external validity of EERW were suspected including within-trial randomization inflating placebo effect, regression to mean, and sensitivity of results on methods.^16^ EERW might fail to contribute to the individualization of diagnosis and reinforces the gap between trials for regulatory approval and clinical practice.^17^ Likewise, it fails to help clinicians in finding the right drug for the patient and concerns on blinding of patients.^18^ Another issue following the OL period is bias in estimating responder proportions within sub-populations.^19^

Placebo induced was also reported due to OL Period before or abrupt change to placebo at randomization.^5^^,^^18^ Finally, an obvious lack of identification of adverse effects was observed.^20^

Previously, these critiques were cited without evaluation of their actual presence or significance. Our aim was to determine their existence and measure their impact using historical data, while proposing an approach to mitigate their influence.

## Material and methods

### Categorization and measurement of effects

Our hypothesis was that any potential bias in an EERW trial could only stem from two unique features: the Open-Label (OL) phase and the subsequent responder selection. We therefore focused on developing proxy measures for the effects of these two features (OLE and SLE) and testing their significance. This effect may manifest as persistence of treatment effects, placebo amplification, increased inter-individual variability, or potential treatment bias. The second is the selection of responders at the end of the open-label phase, which we associate with a Selection Effect (SLE), potentially driven by underlying phenomena such as regression to the mean, inappropriate patient selection, or bias during the Double-Blind (DB) phase.

Rather than hypothesizing the likelihood of these underlying mechanisms, we limited our assumption first in identifying an adequate measurement for each OLE and SLE effect, followed by testing their significance using the results and data of historical EERW trials.

### Proposed measures of OLE and SLE

A key challenge in identifying optimal measures lies not only in their intrinsic validity for quantifying each effect, but also in their availability in historical studies. In the absence of clear guidance from the literature and following a review of publications describing EERW trials as well as informal discussions with colleagues, we adopted the following measures: Let Z denote the primary endpoint of the study, with initial value Zi at the start of the OL phase, baseline Zb at the start of the DB phase, and final value Zf at the end of the DB period for the overall population.

The OLE effect was measured as the change Zb-Zi during the OL period, these values being commonly reported. Although longitudinal data would provide a more accurate assessment of this effect, such data were rarely available.

The SLE effect was estimated using the selection ratio θ = ni/nb, where ni and nb represent the number of patients entering the OL phase and the number entering the DB phase, respectively. Ideally, assessing SLE would require measures of variability around Zb, however, because these are seldom reported, this approach is generally not feasible.

## Material

We conducted a retrospective analysis based on historical EERW trials in compliance with the STROBE guideline.^21^

Data base search was restricted to Cochrane Central Register of Controlled Trials, MEDLINE (OVID, 1966–15 Feb 2025), Embase (OVID, 1980–15 Feb 2025), and LILACS (1982–15 Feb 2025), additional sources included Clinicaltrials.gov, WHO ICTRP, and PROSPERO to find ongoing or unpublished studies. All searches were completed by March 15, 2025.

Keywords: Initial study selection was performed via umbrella research, utilizing meta-analyses and systematic reviews identified by the keywords (‘Meta-analysis’ or ‘systematic review’ and ‘EERW’ or ‘RWT’). An additional selection included studies identified with the keywords (‘Randomized trial’ and ‘EERW’ or ‘RWT’).

The Selection was any EERW trial irrespective of country or language, for which the sample size and the estimate of the main endpoint were available at the start and the end of the OL period, and at end of the DB period.

Data extraction and final summary table detailing these characteristics for each study, was independently completed by three raters (PL, GE, HD), checking for repeated publications on the same data, and discrepancies reconciled subsequently. The reasons for excluding trials were documented in the list of trials.

Extracted data were: authors name; year of publication; the country where the trial was conducted; studied treatment, pathology and classification of pathology; studied main endpoint, sample size and main endpoint for the three important visits (OL initial, OL final, DB final for studied treatment and control).

Study methodological quality was assessed by the same raters using the Revised Cochrane risk-of-bias tool for randomized trials RoB-2,^22^ requiring coding across five main domains: bias from the randomization process, deviations from intended interventions, missing outcome data including participants withdraw from the study or LFU or dropout, measurement of the outcome and selection of the reported result. These items were coded as Low, Unclear, and high. The certainty of the evidence was assessed using the GRADE approach^23^ and summarized by classifying the studies as eligible or rejected.

### Statistical modelling and inference

The analysis was conducted using R statistical software [R; 2012] Statistical significance was defined as p < 0.05. The planned procedures were detailed in a pre-specified statistical analysis plan, which was finalized prior to data collection, and summarized as follows:

To minimize heterogeneity across studies using different and non-directly comparable endpoints, we standardized main endpoints as *Z*-scores by dividing each value by the estimated baseline SD_b_. We used an ANCOVA model, structured as a linear mixed model, with Z_f_ and Z_b_ endpoint estimates at the final visit and baseline during the DB period to which we added OLE and SLE terms measured as defined previously. To address study heterogeneity, we also added publication year as a fixed continuous term and pathology as a random categorical factor, the model admitting a residual term ϵ hypothesized to follow a normal distribution N(0, σ) and summarized as: Zf∼Zb+OLE+SLE+year+{pathology}+ϵ

We first checked the model assumptions in testing normality, homoscedasticity, and collinearity, before fitting the model. Our main objective was to evaluate the effects of OLE and SLE by the significance of the changes in R-squared, AIC, and BIC values when removing them from the model. In a subsequent step, to evaluate the potential biases introduced by SLE and OLE on the treatment effect, we used the dependent variable Δ = (Z_t_-Z_b_) between the treatment group Z_t_ and the control group Z_f_ at the end of the double-blind phase. For sensitivity purposes, we evaluated the risk of bias effect and its interaction with the SLE and OLE effects, in adding these effects in the model.

We finally evaluated the effect of adding OLE and SLE terms on the resulting statistical power of the test of treatment effect, and the required sample size related with an assumed value of the treatment effect, through a formulation depending on the R^2^ determination coefficient of the model (Suppl. 6). Each model fitting results was reported with the estimated coefficient and 95% CI of each term, and its standardized beta coefficient β_std_ enabling a between effect comparison.

## Results

### Study selection

The umbrella search identified 6 meta-analyses or systematic reviews on EERWs, mainly related to drugs for neurological disorders with or without opioids (Table 1). A total of 42 studies were selected, with 39 distinct individual EERW studies extracted from these references to which we added three recent studies on narcoleptic patients.^27^^,^^28^^,^^29^ Out of them, 6 were classified as high risk and did not report the necessary data at initial, baseline, and final time points, thus 36 studies were finally eligible. The studied pathologies were classified as general pain (22), narcolepsy (3), diabetes (4), and other pathologies (7).

**Table 1:** List of meta-analyses and/or systematic research identified in the literature, the studied pathology, and the number of used studies.

Study	Number of studies	Topic
Meske^24^	15	Opioids in chronic pain
Furlan^10^	8	Opioids noncancer in chronic pain
Katz^4^	29	Opioids for chronic pain
Kopsky^25^	28	Chronic pain
McMillan^26^	18	Opioids for chronic pain
Moore^18^	25	Chronic pain

We include a general overview of each file (Suppl. Table 1) and provide raw data from each study used in the analysis (Suppl. Table 2). Most trials were dated, and aside from publication year and disease studied, some notes specific to each study are provided. Endpoint summary values at initial, baseline, and final times were standardized and linearly transformed so that all endpoints decrease as patients improve. An overall mean initial value of 7.85±3.19 decreased to 3.13±0.93 at the end of the OL period, followed by an ascent to 6.08±2.48 (control group) and 4.12+3.1 (studied treatment group). The OLE and SLE estimates were 4.72±2.95, and 0.62±0.19, respectively (Table 2).

**Table 2:** Descriptive statistics of the standardised values at initial (Zi), baseline (Zb), placebo (Zf), and studied treatment (Zt) final timepoints.

	Mean	± SD	Median	[Q1,	Q3]
Z_i_	**7.85**	± 3.19	**7.46**	[5.29,	10.04]
Z_b_	**3.13**	± 0.93	**3.04**	[2.42,	3.51]
Z_f_	**6.08**	± 2.48	**5.54**	[4.51,	6.25]
Z_t_	**4.12**	± 3.10	**3.40**	[2.39,	4.55]
OLE	**4.72**	± 2.95	**3.87**	[2.52,	6.42]
SLE	**0.62**	± 0.19	**0.63**	[0.52,	0.69]

### Assessing the effects of OLE and SLE

We first assessed the underlying assumptions of our model: homoscedasticity was concluded for each covariate (Levene test) and within group errors were distributed according to a normal distribution (Shapiro Test, p > 0.05). Collinearity was ruled out, as the tolerance values (the inverse of the Variance Inflation Factor) for all covariates ranged from 0.8 to 1.4 with a condition number CN = 4.23. A weak and non-significant was found between OLE and SLE measures (*r* = 0.181, 95% CI [−.081, 0.444], p = 0.177).

The linear fit on final Z_f_ (Table 3) was characterized by a adjusted determination coefficient R^2^ = 0.591, it showed highly significant effects (p < 0.001) for baseline adjustment Z_b_ (1.126 [0.546, 1.706], β_std_ = 0.422), for the OLE effect (0.477 [0.280, 0.673], β_std_ = 0.567), followed by a significant SLE effect (3.396 [0.509, 6.284], p = 0.028, β_std_ = 0.263), and no significant effect of publication year (−0.088 [−0.181, 0.003], p = 0.098) or random pathology (estimated between pathology SD = 0.632, p = 0.537).

**Table 3:** Linear mixed model assessing the significance of baseline Z_b_, placebo-inflated effect OLE, and SLE to the mean effect SLE on the final endpoint Z_f_ at the end of the DB Period.

	Effect	95%	CI	Standardised β_std_	*P*-value
Overall estimate	6.078	5.557,	6.599	-	**<.001**
Z_b_	1.126	0.546,	1.706	.422	**0.001**
OLE effect	0.477	0.280,	0.673	.567	**<.001**
SLE effect	3.396	0.509,	6.284	.263	**0.028**
Publication Year	−0.088	−0.181,	0.003	-.217	**0.068**

Note: Studied pathology was assimilated as a random factor.

The relevance of adding OLE and SLE effects in the ANCOVA model is confirmed by smaller AIC (164 vs. 144), BIC (169 vs. 152), and a highly statistically significant increase of the R^2^ determination coefficient (p < 0.001) (Table 4).

**Table 4:** Comparison of Model 1 (adjusting for SLE and OLE) with Model 0 (simple ANCOVA only adjusting for baseline Z_b_).

Model	Degree fredom	R^2^	AIC	BIC	ΔR^2^*P*-value
ANCOVA	4	.211	164	169	-
Adding OLE, SLE	6	.591 Δ=.380	144	152	<.001

AIC, Akaike Information Criterion, BIC, Bayes Information criterion, ΔR^2^, increase of the determination coefficient, observed in adding OLE and SLE effects.

The studied treatment effect was estimated by the difference Δ = (Z_t_-Z_c_) between the means in the treatment (Z_t_) and the control (Z_c_) subgroups that was shown non-significantly influenced by OLE (−0.185 [−0.060, 0.430], p = 0.150) and SLE (−1.703 [−1.914, 5.321], p = 0.363) (Suppl. Table 5).

For sensitivity purposes, we showed the non-significance of the methodological quality main effect, and its interaction with SLE and OLE effects (Suppl. Table 3), and the non-significant interaction between the SLE and OLE effects (Suppl. Table 4).

To emphasize practical implication of these results, Table 5 evaluates the effect of adding OLE and SLE terms in the ANCOVA model on the statistical power in assuming a true standardized mean difference of 0.5, two-sided test significance α = 0.05, for some determined sample size per group, and the needed sample size for usual values of the power.

**Table 5:** Power and sample size per treatment group corresponding to SMD = 0.5.

		Power for sample size:				Sample size for power:			
	R^2^	20	30	40	50	0.99	0.95	0.9	0.8
**ANCOVA**	0.211	0.429	0.587	0.712	0.804	116	82	66	50
**+OLE, SLE**	0.591	0.696	0.857	0.938	0.974	60	43	34	26

## Discussion

### Evidence of a specific effect of EEWR trial and potential causes

Compared to Randomized Controlled Trials (RCTs), Enriched Enrolment Randomized Withdrawal (EERW) trials have two main features: an open-label phase and the patient selection process at its conclusion. Our reasoning was that criticism documented in literature arises from these particular aspects. Instead of exploring the root causes, we decided to hypothesize the existence of two related potential effects and identify a measurable parameter for each, which can be assessed through historical studies.

Our primary objective was to evaluate potential biases in treatment effect estimates and the resulting loss of precision. Furthermore, we aimed to determine how appropriate statistical analyses could mitigate these issues. Having established the presence of these effects, we now discuss the putative reasons for these findings:

The Open-Label Extension (OLE) effect was highly significant. Specifically, the OL period resulted in baseline values that differed substantially from those observed in a Randomized Controlled Trial (RCT). Given that baseline values necessitate major adjustments to the final endpoint in nearly all trials, this impact on the outcome is expected; the magnitude of the effect increased in proportion to the difference Zb-Zi used to measure the OLE effect.

Furthermore, these elevated values exhibited high inter-patient variability. This discrepancy and its associated variance provide a plausible explanation for the OLE effect. Other contributing factors may include carry-over effects, particularly following drug withdrawal. For instance, a pharmacologically active but therapeutically ineffective drug may appear superior to placebo due to withdrawal symptoms or dependency developed during the OL treatment period.^18^

The Selection (SLE) effect, quantified by the selection ratio θ = nb/ni was also identified as a significant predictor of the final endpoint estimate. This phenomenon can be attributed to several factors, most notably the uncertainty in responder selection caused by the regression to the mean of the endpoint at the conclusion of the Open-Label (OL) phase and is further compounded by the substantial inter-patient variability induced during this phase.

A higher recruitment coefficient is associated with an increased final endpoint score. In this dataset (Table 3), where lower scores indicate clinical improvement, the positive SLE coefficient reflects a trend toward worsening outcomes as the recruitment threshold becomes more restrictive. This suggests that while a stricter selection threshold identifies better responders for the treatment phase, it also introduces a selection bias that shifts the final outcome. Consequently, the apparent treatment effect is influenced more by the stringency of the lead-in criteria than by the drug's inherent efficacy.

Finally, the clinical relevance of these effects can be evaluated using standardized β_std_ coefficients, which allow for a comparison of covariate importance within the model (Table 3). Although baseline adjustment is an essential component of an ANCOVA model, the OLE effect yielded a higher β_std_ coefficient (0.567) than the baseline effect (0.422), while the SLE effect produced a comparatively smaller β_std_ (0.263). In terms of clinical impact, these standardized β_std_ coefficients are analogous to the standardized mean difference: Applying Cohen’s thresholds,^30^ a value exceeding 0.2 is considered a minimum clinically relevant effect, while a value greater than 0.5 indicates a medium effect size.

As a last note, we provide evidence of robustness of our findings on OLE and SLE effects in showing that these effects were independent of the methodological quality main effect, and its interaction with SLE and OLE effects (Suppl. Table 3).

### Consequences for the analysis of a EERW

Our results show that OLE has a stronger impact than baseline effects, making it the main source of unnecessary residual variability, which in turn prevents to accurately assess treatment effects. Including these effects in the ANCOVA model reduces the overall residual variability and helps correct potential imbalances between the two groups.

Another important finding is that, despite their undesired effects on residual variability, no bias is expected in testing the significance of the studied treatment effect at first glance, this was a priori expectable owing that OLE and SLE are baseline pre-randomized data, thus assimilable to baseline covariates.

### Power gain

The statistical power of EERW designs has been a subject of ongoing debate. Some authors suggest that the increased sample homogeneity resulting from the Open-Label phase may enhance statistical power. Conversely, recent findings have challenged this assumption; for instance,^26^ reported that a non-EERW study utilizing missing data imputation yielded more significant results than comparable EERW designs. Regardless of these findings, we question the relevance of directly comparing the power of EERW and Randomized Controlled Trial (RCT) designs. Comparing these designs within the same population or disorder is of limited value due to fundamental differences in the target populations. Therefore, a meta-analytical unconditional pooled estimate of EERW and non-EERW trials is problematic unless a meta-regression is employed to quantify the design effect. Ultimately, these two designs yield non-comparable results, rendering direct comparisons invalid. Our objective was not to address the fundamental interpretative differences between EERW and RCT designs. Rather, we provide evidence that even if EERW results are accepted as valid, the design inherently introduces substantial residual variability.

### Implication for practice

Our proposal is straightforward and easily implemented: a) Both the OLE and SLE effects are easily quantifiable during the Open-Label (OL) phase prior to randomization; they are fully justified as pre-Randomization adjustments, and the standard statistical framework remains unchanged except for the inclusion of these two effects as covariates. b) While OLE and SLE are theoretically expected to be balanced across treatment arms, our approach ensures that any minor stochastic imbalances between groups are effectively accounted for and corrected.

### Limitations

Significant criticism has been directed toward the relevance and applicability of EERW designs, particularly regarding the interpretation of results and limited external validity. The scope of this study was restricted to specific biases inherent in the design and the potential to mitigate them through statistical adjustment. It is not suggested that our proposal addresses all methodological concerns associated with EERW designs. However, the resulting increase in precision ‒ often cited as the primary advantage of this design ‒ helps alleviate some well-known critiques.

Several important methodological limitations should be noted. First, the small number of studies (n = 36) leads to statistical uncertainty, as reflected in the wide confidence intervals. This is a common challenge in meta-regression analyses based on published summary statistics and can only be fully addressed through individual patient data meta-analyses, which are unfeasible here due to the heterogeneity of sponsors and authors in older studies.

Second, the development of our proxy measures was constrained by the level of detail available in source publications. While measuring OLE via the change Zb-Zi proved effective, a more precise SLE estimation would require repeated intermediate OL period estimates, which are seldom reported. Furthermore, while the selection ratio θ is a useful metric for meta-analysis, it is not applicable to a single study.

Third, the generalizability of our findings is supported by the analysis of therapeutic areas and methodological quality; no significant interactions were observed between these variables and the OLE or SLE effects. However, it cannot be definitively confirmed that this set of historical studies is fully representative of the entire EERW population.

## Conclusion

Although EERW designs are widely utilized, their methodology remains a subject of ongoing debate. In this study, effects arising from the Open-Label (OL) phase and the subsequent selection of patients have been categorized as OLE and SLE effects, respectively. We have attempted to quantify these phenomena, demonstrating that while they may not directly bias the treatment effect estimate, they significantly increase residual variability. By incorporating these two effects as adjustments within the inferential model, this extraneous variability can be mitigated, leading to more precise and robust statistical outcomes.

## Authors’ contributions

Philippe Lehert is the sole author of this study and performed the Conceptualization, Methodology, Validation, Formal analysis, Investigation, Resources, Data management, Writing, Visualization, Supervision, Project administration. There was no Funding.

## Data availability

The datasets generated and/or analyzed during the current study are available from the corresponding author upon reasonable request.

## Declaration of competing interest

The authors declare no conflicts of interest.
