# Supplementary material for: Methodological considerations for analyzing enriched enrolment randomized withdrawal trials
Source: Clinics (Sao Paulo). 2026 Sep 9;81:101131. doi: 10.1016/j.clinsp.2026.101131 (PMC13573709; doi:10.1016/j.clinsp.2026.101131)
Supplement: Supplementary file 1 [file mmc1.docx]

CLINICS-D-26-00021_Supplementary Matertial

**Supplement**

**Supplement Table 1** This table summarises the data collection from the 42 studies.

|  | **Study** | **Treatment** | **PC** | **Indication ‒ Pathology** | **Endpoint** | **Notes** |
| --- | --- | --- | --- | --- | --- | --- |
| 1 | Arai1[1] | Fentanyl | 1 | Chronic Pain | Pain VAS | Change of 0.2 (18.21) and -6.9 (20.84) converted in final value. |
| 2 | Arai2[1] | Fentanyl | 1 | Chronic Pain | PAIN VAS | Changes were similarly provided in secondary endpoints and converted. |
| 3 | Arnold[2] | Pregabaline | 0 | Fibromyalgia | Relapse | Abstract (S. Size), Figure 4 (change in time). |
| 4 | Baron[3] | Pregabalin | 1 | Chronic Pain | Pain score | Change provided and converted in final value. |
| 5 | Bogan[4] | Sodium oxybate | 0 | Narcolepsy | nCTP | Selection (Fig. 2) mean values Fig. 3 |
| 6 | Burch[5] | Tramadol | 0 | Osteoarthritis | PI-NRS | Tramadol vs. Pcb. Pain Intensity-Numerical Score (PI-NRS). During the RCT, values are mean changes and not final values. Final values were recalculated. |
| 7 | Byas-Smith[6] | Clonidine | 3 | Diab | Pain | Crossover design Figure 1 with approximation. |
| 8 | Caldwell[7] | Oxycodone | 0 | Arthritis | PI | Pain intensity 0‒3. |
| 9 | Clauw[8] | Minacipram | 0 | Fibromyalgia | VAS PAIN | VAS, Δ=50%. Results found on Figure 3 providing LS-mean changes. These have been converted into final values. |
| 10 | Coghill[9] | Pregabaline | 1 | ADHD | ADHD score | Initial values found in Table 1, and ADHD values in efficacy text. |
| 11 | Crofford[10] | Pregabalin | 0 | Fibromyalgia | Pain VAS | Not provided VAS value at baseline. |
| 12 | Cyndia[11] | Orthos | 1 | Hypotension | OL | Question Occupation health questionnaire OHQ, value of OL in the table on p104, and change in time only found on Figure 3. |
| 13 | Dauvilliers[12] | Sodium oxybate | 2 | Somnolence | ESS | Only the difference is provided: -6.5 between the 2 groups. |
| 14 | Friedmann[13] | Oxycodone | 0 | Arthritis | AUC pain | Figure 3, Table 1. AUC thus suppose change from Initial -3.4 and -5.6 |
| 15 | Galer[14] | Lidocaine | 1 | Neuralgia | Pain stop | Only percent’s for the main endpoint were found. Data non compatible with the selection |
| 16 | Hale 3[15] | Hydrocodone | 0 | Back Pain | Pain | Δ=patient reaching Optimal dose. |
| 17 | Hale1[16] | Oros | 0 | Back Pain | Pain score | Pain score PI-NRS. Results from Figure 3. Δ: multiple conditions to enter into the RCT; not possible to summarise by a Δ-value. |
| 18 | Hale2[17] | Hydrocodone | 0 | Back Pain | Pain score | Average Pain Intensity 0‒10. Δ not identifiable. |
| 19 | Huffman[18] | Pregabalin | 0 | Neuralgia | Pain | Average Pain Intensity 0‒10. |
| 20 | Katz1[19] | Morphine | 0 | Osteoarthritis | Pain score | Brief Pain Inventory 0‒10. Δ<4 and declined >2. |
| 21 | Katz2[20] | Oxymorphone | 0 | Back Pain | Pain | VAS 0‒100 |
| 22 | Kawamata[21] | Oxycodone | 0 | Back Pain | Pain | Japan. No scale was used. Only the proportion of patients controlled. |
| 23 | Landau[22] | Buprenorphine | 0 | N-cancer Pain | Pain | Endpoint = % of patients-controlled baseline, and final OL values not available. |
| 24 | Lynch[23] | Adenosine | 0 | Neuropathy | VA pain | VAS; Necessary values are not available |
| 25 | Plazzi[24] | Sodium oxybate | 2 | Narcolepsy | N cataplexy attacks | Number, n, of cataplexy attacks; I received the initial data from the author. |
| 26 | Portenoy[25] | Fentanyl | 0 | Back Pain | Pain score | Summed Pain Intensity at 60-min. Values not directly comparable and converted. |
| 27 | Raskin[26] | Pregabalin | 3 | Diabetic Neuropathy | Pain Score | Directly estimated from Figure 2 (sample) and Table 1 |
| 28 | Ratain[27] | Sorafenib | 1 | Carcinoma | Survival | No statistics available for this study compatible with our selection. |
| 29 | Rauck 2014[28] | Hydrocodone | 0 | Back Pain | Pain score | Figure 2 (patients), Table 4 for mean changes converted into final values. |
| 30 | Rauck 2015[29] | Oxycodone | 0 | Back Pain | Pain score | Data found from graphical interpretation of Figure 2 (patients), Figure 3 (endpoints) |
| 31 | Schnitzer-1[30] | Tramadol | 0 | Arthritis | Pain VAS | Naproxen responders: only values for end of OL. Calibrated with the main endpoint. |
| 32 | Schnitzer-2[31] | Tramadol | 0 | Arthritis | Pain VAS | Naproxen non-responders: Only values for end of OL. Not used as no conformity with EERW trial. |
| 33 | Schwarz[32] | Tapentadol | 3 | Diabetic Neuropathy | Pain Sc | Data found from graphical interpretation from Figure 1 (sample size) and Figure 2 (change of Pain Score). |
| 34 | Simpson[33] | Fentanyl | 0 | Neur Pain | Pain score | (SPId60) crossover. Fig. 3 and Table 1. Fig. 3 interpreted as change from Baseline. |
| 35 | Steiner[34] | Buprenorphin | 0 | Back Pain | Pain Sc | Results from Figure 2 (sample size), Table 2 (Pain) and Table 3. |
| 36 | Strollo[35] | Solriamfetol | 2 | Somnolence | ESS | ESS Initial found in Table 1, mean of columns 3 and 4. ESSb and ESSf found on Figure 3. SD not provided; but estimated from the graphic and approximated by their initial values. |
| 37 | Tandon[36] | Lurasidone | 1 | Schizophrenia | PANSS | No SD reported. Values during the OL is the PANSS scale found. Yi found in the text pp 72 bottom; Probability of relapse found in pp 73 Alinea 3. |
| 38 | Vinik[37] | Tapentadol | 3 | Diabetic Neuropathy | Pain Sc | Data found from graphical interpretation Results Fig. 1 (sample size) and Fig. 2 (Pain Scores). |
| 39 | Vondrackova[38] | Oxycodone | 0 | Chronic Pain | BowIndex | Only sample sizes are documented; Table 2 provides the main statistics. No initial values are available. Study excluded. |
| 40 | Vorsanger[39] | Tramadol | 0 | Back Pain | Pain VAS | Data found from graphical interpretation Fig. 2 (sample size), efficacy section 2e Alinea + Figure. 3 |
| 41 | Wen[40] | Hydrocodone | 0 | Back Pain | Pain Sc | Figure 2 (sample size), Figure 3 (efficacy). |
| 42 | Zakrzewska[41] | NA blocker | 1 | Trigeminal Neuralgia | Pain VAS | Data found from graphical interpretation Figure 1 (sample size), Figure 3 (Pain score) Initial value unknown ≅70 |

ADHD, Attention-Deficit/Hyperactivity Disorder; PANSS, Positive and Negative Syndrome Scale; VAS, Visual Analogue Scale.

Note: The list of the publications is provided in section 7.3. PC is the pathology category regrouped into 0 = Pain, 2 = Narcolepsy/Stimulants, 3 = Diabetic Neuropathy, 1 = Other.

**Supplement Table 2** Main scores and main characteristics of each study. The OL phase starts with an initial Z_i_ observed value of the main endpoint, the final observed value of OL phase is noted as Z_b_ (which is also the baseline value of the DB phase), and the final value of the DB noted Z_f_ for placebo group and Z_t_ for the studied treatment group. Ni and Nb are the sample size at initial and baseline time. PC is pathology.

| **Num** |  | **Year** | **Zi** | **Zb** | **Zf** | **Zt** | **Nb** | **Ni** | **Lim** | **Sel** | **Pc** |
| --- | --- | --- | --- | --- | --- | --- | --- | --- | --- | --- | --- |
| 1 | Arai1[1] | 2015 | 9.13 | 3.61 | 5.11 | 4.79 | 150 | 218 | 0.5 | 1 | 3 |
| 2 | Arai2[1] | 2015 | 8.95 | 3.51 | 5.03 | 3.13 | 163 | 258 | 0.5 | 1 | 3 |
| 3 | Arnold[2] | 2014 | 11.12 | 5.23 | 5.72 | 5.72 | 121 | 441 | 0.5 | 1 | 0 |
| 4 | Baron[3] | 2009 | 11.68 | 4.22 | 5.81 | 4.29 | 217 | 378 | 0.5 | 1 | 3 |
| 5 | Bogan[4] | 2021 | ‒ | ‒ | ‒ | ‒ | 136 | 201 | 0.5 | 0 | 1 |
| 6 | Burch[5] | 2007 | 6.58 | 4.99 | 8.08 | 7.07 | 646 | 1028 | 2.0 | 1 | 0 |
| 7 | Byas-Smith[6] | 1995 | 3.99 | 3.33 | 5.49 | 3.33 | 12 | 41 | 0.3 | 1 | 2 |
| 8 | Caldwell[7] | 1999 | 5.53 | 2.21 | 5.92 | 0.66 | 107 | 167 | 0.5 | 1 | 0 |
| 9 | Clauw[8] | 2013 | 8.33 | 2.43 | 4.22 | 2.28 | 151 | 347 | 0.5 | 1 | 0 |
| 10 | Coghill[9] | 2014 | 11.89 | 3.03 | 8.84 | 3.60 | 177 | 276 | ‒ | 1 | 3 |
| 11 | Crofford[10] | 2008 | 10.92 | 1.68 | 3.57 | 2.11 | 566 | 1051 | 0.3 | 1 | 0 |
| 12 | Cyndia[11] | 2015 | 10.27 | 3.05 | 7.60 | 6.42 | 151 | 181 | ‒ | 1 | 3 |
| 13 | Dauvilliers[12] | 2022 | 5.73 | 2.23 | 4.97 | 2.45 | 115 | 154 | ‒ | 1 | 1 |
| 14 | Friedman[13] | 2011 | 4.94 | 3.51 | 4.23 | 1.30 | 412 | 558 | 0.3 | 1 | 0 |
| 15 | Galer[14] | 1999 | ‒ | ‒ | ‒ | ‒ | 32 | ‒ | ‒ | 0 | 3 |
| 16 | Hale1[15] | 2010 | 6.24 | 3.12 | 5.40 | 3.22 | 268 | 459 | ‒ | 1 | 0 |
| 17 | Hale2[16] | 2007 | 4.14 | 2.54 | 4.10 | 2.60 | 294 | 519 | ‒ | 1 | 0 |
| 18 | Hale3[17] | 2015 | 4.90 | 2.57 | 4.56 | 2.63 | 271 | 625 | ‒ | 1 | 0 |
| 19 | Huffman[18] | 2017 | 3.96 | 1.83 | 2.98 | 0.89 | 413 | 801 | 0.5 | 1 | 0 |
| 20 | Katz1[19] | 2010 | 7.65 | 3.50 | 6.04 | 3.89 | 344 | 547 | 2.0 | 1 | 0 |
| 21 | Katz2[20] | 2015 | 9.81 | 2.57 | 7.75 | 4.17 | 205 | 325 | 0.0 | 1 | 0 |
| 22 | Kawamata[21] | 2019 | ‒ | ‒ | ‒ | ‒ | 130 | 189 | 0.0 | 0 | 0 |
| 23 | Landau[22] | 2007 | ‒ | ‒ | ‒ | ‒ | 267 | 588 | 0.0 | 0 | 0 |
| 24 | Lynch[23] | 2003 | ‒ | ‒ | ‒ | ‒ | 40 | 66 | 0.3 | 0 | 0 |
| 25 | Plazzi[24] | 2018 | 16.28 | 3.48 | 10.49 | 5.43 | 63 | 136 | 0.0 | 1 | 1 |
| 26 | Portenoy[25] | 2007 | 4.79 | 3.38 | 4.97 | 7.79 | 77 | 124 | ‒ | 1 | 0 |
| 27 | Raskin[26] | 2014 | 5.99 | 2.86 | 4.09 | 2.86 | 294 | 665 | 0.3 | 1 | 2 |
| 28 | Ratain[27] | 2001 | ‒ | ‒ | ‒ | ‒ | 33 | 202 | 0.2 | 0 | 3 |
| 29 | Rauck14[28] | 2014 | 8.64 | 3.83 | 5.94 | 5.06 | 102 | 510 | 0.5 | 1 | 0 |
| 30 | Rauck15[29] | 2015 | 7.86 | 3.32 | 5.48 | 4.32 | 279 | 407 | 0.5 | 1 | 0 |
| 31 | Schnitzer1[30] | 1999 | 7.97 | 1.84 | 5.92 | 2.21 | 90 | 155 | ‒ | 1 | 0 |
| 32 | Schnitzer2[31] | 1999 | 5.57 | 2.99 | 6.32 | 3.37 | 146 | 210 | ‒ | 1 | 0 |
| 33 | Schwartz[32] | 2011 | 4.86 | 2.39 | 4.44 | 2.26 | 395 | 591 | 1.0 | 1 | 2 |
| 34 | Simpson[33] | 2007 | 13.80 | 5.31 | 16.09 | 19.10 | 79 | 102 | 0.2 | 1 | 0 |
| 35 | Steiner[34] | 2011 | 7.82 | 3.26 | 6.27 | 4.14 | 541 | 1027 | 0.3 | 1 | 0 |
| 36 | Strollo[35] | 2019 | 11.25 | 4.53 | 6.18 | 7.89 | 62 | 124 | ‒ | 1 | 1 |
| 37 | Tandon[36] | 2015 | 3.60 | 2.17 | 4.46 | 2.32 | 285 | 676 | ‒ | 1 | 3 |
| 38 | Vinik[37] | 2014 | 5.44 | 2.54 | 5.13 | 2.90 | 320 | 459 | 0.0 | 1 | 2 |
| 39 | Vondrackova[38] | 2008 | 7.27 | 4.09 | 6.23 | 4.18 | 464 | 751 | 0.3 | 1 | 0 |
| 40 | Vorsanger[39] | 2008 | 6.55 | 2.95 | 5.58 | 3.43 | 386 | 619 | 0.0 | 1 | 0 |
| 41 | Wen[40] | 2015 | 5.14 | 2.11 | 4.25 | 2.32 | 588 | 905 | 0.3 | 1 | 0 |
| 42 | Zakrzewska[41] | 2017 | 14.07 | 2.41 | 11.55 | 4.02 | 29 | 44 | 0.3 | 1 | 3 |

Note: The 4 values were standardised (divided by the SD_b_ standard deviation) for inter-study comparability. Lim was the threshold used for classifying patients as responders. This table was independently completed by 2 raters. Decreasing values of the endpoint correspond to patient improvement. For studies using the opposite direction, the values were reversed. The PC column is pathology group: 0=Pain; 1=Narcolepsy, OSA; 2=Other neurological disorders; 3=Other pathologies. SEL=eligibility of studies: 0=noneligible due to important missing data.

.

**Supplementary Table 3** Linear model assessing the effect of methodological quality and its interaction with SLE and OLE on the final endpoint (Yf).

|  | **Coef** | **Se** | **95%** | **CI** | **p-val** |
| --- | --- | --- | --- | --- | --- |
| **Intercept** | 6.126 | 0.282 | 5.573, | 6.679 | <0.001 |
| **Zb** | 1.043 | 0.322 | 0.412, | 1.673 | 0.003 |
| **Ole** | 0.507 | 0.111 | 0.290, | 0.723 | <0.001 |
| **Sle** | 3.140 | 1.609 | -0.014, | 6.294 | 0.061 |
| **Quality** | 0.414 | 0.931 | -1.411, | 2.239 | 0.660 |
| **Year** | -0.074 | 0.074 | -0.220, | 0.071 | 0.322 |
| **Ole:quality** | 0.219 | 0.264 | -0.299, | 0.737 | 0.415 |
| **Sle:quality** | -0.542 | 3.767 | -7.926, | 6.842 | 0.886 |

**Supplementary Table 4** Linear model assessing the interaction of SLE and OLE in addition to their main effects on the final endpoint (Yf).

|  | **Coef** | **Se** | **95%** | **CI** | **p-val** |
| --- | --- | --- | --- | --- | --- |
| **Intercept** | 5.969 | 0.266 | 5.447, | 6.490 | <0.001 |
| **Zb** | 1.132 | 0.287 | 0.569, | 1.694 | <0.001 |
| **Ole** | 0.422 | 0.102 | 0.222, | 0.623 | <0.001 |
| **Sle** | 3.193 | 1.435 | 0.380, | 6.005 | 0.034 |
| **Year** | -0.084 | 0.046 | -0.175, | 0.005 | 0.073 |
| **Ole:sle** | 0.752 | 0.442 | -0.114, | 1.619 | 0.099 |

**Supplementary Table 5** Linear model assessing the studied treatment effect estimated by the difference Δ =(Z_t_-Z_c_) between the means in the treatment (Z_t_) and the control (Z_c_) subgroups.

|  | **Coefficient** | **SE** | **95%** | **CI** | **p-value** |
| --- | --- | --- | --- | --- | --- |
| **Intercept** | 1.963 | 0.334 | 1.307, | 2.619 | <0.001 |
| **Baseline Z_b_** | 0.017 | 0.025 | -0.030, | 0.066 | 0.486 |
| **OLE effect** | 0.185 | 0.125 | -0.060, | 0.430 | 0.150 |
| **SLE effect** | 1.703 | 1.845 | -1.914, | 5.321 | 0.363 |
| **Publication year** | -0.035 | 0.060 | -0.152, | 0.081 | 0.553 |

**Supplement 6** Statistical power and sample size calculation based on determination coefficient.

1. Statistical Power Formula [Frison, Pocock, 1992]

The power ($1-\beta$) for testing the treatment effect in an ANCOVA model can be approximated using the non-central $F$-distribution or, more commonly for sample size planning, the standard normal distribution:

$$Power=\Phi\left( \sqrt{\frac{N\cdot f^{2}}{1-R^{2}}}-z_{1-\alpha/2} \right)$$

Where: $\Phi$ is the cumulative distribution function of the standard normal distribution; $N$ is the total sample size; $f^{2}$ is Cohen’s effect size ($f^{2}=\frac{\delta^{2}}{4\sigma^{2}}$ for equal allocation, where $\delta$ is the true treatment effect); $R^{2}$ is the proportion of variance in $Z_{f}$ explained by $Z_{b}$ and other covariates; $z_{1-\alpha/2}$ is the critical value for the significance level $\alpha$.

2. Sample Size Calculation Formula [Borm et al, 2007]

To calculate the required sample size per group ($n$) to achieve a specific power, we use the the formula: $n=\frac{2\sigma^{2}\left( z_{1-\alpha/2}+z_{1-\beta} \right)^{2}}{\delta^{2}}\cdot\left( 1-R^{2} \right)$

Variables Breakdown: $\sigma$: The standard deviation of the endpoint $Z_{f}$ (unadjusted); $\delta$: The assumed true treatment effect (difference in means); $R^{2}$: The coefficient of determination; $z_{1-\alpha/2}$: The z-score for the confidence level (e.g., 1.96 for 95%); $z_{1-\beta}$: The z-score for the desired power (e.g., 0.84 for 80%).

In both expressions, $R^{2}$ represents the correlation between the baseline variables and the post-treatment measure. The term $\left( 1-R^{2} \right)$ acts as a “Variance Inflation Factor” (or rather, a reduction factor), showing exactly how much the sample size can be reduced by including those covariates.

Frison, L., & Pocock, S. J. (1992). Repeated measures in clinical trials: Analysis using mean summary statistics and its implications for design. Statistics in Medicine, 11(13), 1685-1704.

Borm, G. F., Fransen, J., & Lemmens, W. A. (2007). A simple sample size formula for analysis of covariance in randomized clinical trials. Journal of Clinical Epidemiology, 60(12), 1234-1238.

**References**

1. Arai T, Kashimoto Y, Ukyo Y, Tominaga Y, Imanaka K. Two placebo-controlled, randomized withdrawal studies to evaluate the fentanyl 1 day patch in opioid-naïve patients with chronic pain. Curr Med Res Opin. 2015;31(12):2207-18.

2. Arnold LM, Arsenault P, Huffman C, Patrick JL, Messig M, Chew ML, et al. Once daily controlled-release pregabalin in the treatment of patients with fibromyalgia: a phase III, double-blind, randomized withdrawal, placebo-controlled study. Curr Med Res Opin. 2014;30(10):2069-83.

3. Baron R, Freynhagen R, Tölle TR, Cloutier C, Leon T, Murphy TK, et al. The efficacy and safety of pregabalin in the treatment of neuropathic pain associated with chronic lumbosacral radiculopathy. Pain. 2010;150(3):420-7.

4. Bogan RK, Thorpy MJ, Dauvilliers Y, Partinen M, Del Rio Villegas R, Foldvary-Schaefer N, et al. Efficacy and safety of calcium, magnesium, potassium, and sodium oxybates (lower-sodium oxybate [LXB]; JZP-258) in a placebo-controlled, double-blind, randomized withdrawal study in adults with narcolepsy with cataplexy. Sleep. 2021;44(3):zsaa206.

5. Burch F, Fishman R, Messina N, Corser B, Radulescu F, Sarbu A, et al. A comparison of the analgesic efficacy of Tramadol Contramid OAD versus placebo in patients with pain due to osteoarthritis. J Pain Symptom Manage. 2007;34(3):328-38.

6. Byas-Smith MG, Max MB, Muir J, Kingman A. Transdermal clonidine compared to placebo in painful diabetic neuropathy using a two-stage ‘enriched enrollment’ design. Pain. 1995;60(3):267-74.

7. Caldwell JR, Hale ME, Boyd RE, Hague JM, Iwan T, Shi M, et al. Treatment of osteoarthritis pain with controlled release oxycodone or fixed combination oxycodone plus acetaminophen added to nonsteroidal antiinflammatory drugs: a double blind, randomized, multicenter, placebo controlled trial. J Rheumatol. 1999;26(4):862-9.

8. Clauw DJ, Mease PJ, Palmer RH, Trugman JM, Wang Y. Continuing efficacy of milnacipran following long-term treatment in fibromyalgia: a randomized trial. Arthritis Res Ther. 2013;15(4):R88.

9. Coghill DR, Banaschewski T, Lecendreux M, Johnson M, Zuddas A, Anderson CS, et al. Maintenance of efficacy of lisdexamfetamine dimesylate in children and adolescents with attention-deficit/hyperactivity disorder: randomized-withdrawal study design. J Am Acad Child Adolesc Psychiatry. 2014;53(6):647-57.e1.

10. Crofford LJ, Mease PJ, Simpson SL, Young JP Jr, Martin SA, Haig GM, et al. Fibromyalgia relapse evaluation and efficacy for durability of meaningful relief (FREEDOM): a 6-month, double-blind, placebo-controlled trial with pregabalin. Pain. 2008;136(3):419-31.

11. Cyndia Shibao A, Palma JA, Celedonio JE, Martinez J, Kaufmann H, Biaggioni I. Predictors of the pressor response to the norepinephrine transporter inhibitor, atomoxetine, in neurogenic orthostatic hypotension. Hypertension. 2021;78(2):413-22.

12. Dauvilliers Y, Arnulf I, Foldvary-Schaefer N, Morse AM, Šonka K, Thorpy MJ, et al. Safety and efficacy of lower-sodium oxybate in adults with idiopathic hypersomnia: a phase 3, placebo-controlled, double-blind, randomised withdrawal study. Lancet Neurol. 2022;21(1):53-65.

13. Friedmann N, Klutzaritz V, Webster L. Efficacy and safety of an extended-release oxycodone (Remoxy) formulation in patients with moderate to severe osteoarthritic pain. J Opioid Manag. 2011;7(3):193-202.

14. Galer BS, Rowbotham MC, Perander J, Friedman E. Topical lidocaine patch relieves postherpetic neuralgia more effectively than a vehicle topical patch: results of an enriched enrollment study. Pain. 1999;80(3):533-8.

15. Hale M, Khan A, Kutch M, Li S, Hsu C, Xiang Q, et al. Once-daily OROS hydromorphone ER compared with placebo in opioid-tolerant patients with chronic low back pain. Curr Med Res Opin. 2010;26(6):1505-18.

16. Hale ME, Ahdieh H, Ma T, Rauck R. Efficacy and safety of OPANA ER (oxymorphone extended release) for relief of moderate to severe chronic low back pain in opioid-experienced patients: a 12-week, randomized, double-blind, placebo-controlled study. J Pain. 2007;8(2):175-84.

17. Hale ME, Laudadio C, Yang R, Narayana A, Malamut R. Efficacy and tolerability of a hydrocodone extended-release tablet formulated with abuse-deterrence technology for the treatment of moderate-to-severe chronic pain in patients with osteoarthritis or low back pain. J Pain Res. 2015;8:623-36.

18. Huffman CL, Goldenberg JN, Weintraub J, Sanin L, Driscoll J, Yang R, et al. Efficacy and safety of once-daily controlled-release pregabalin for the treatment of patients with postherpetic neuralgia: a double-blind, enriched enrollment randomized withdrawal, placebo-controlled trial. Clin J Pain. 2017;33(7):569-78.

19. Katz N, Hale M, Morris D, Stauffer J. Morphine sulfate and naltrexone hydrochloride extended release capsules in patients with chronic osteoarthritis pain. Postgrad Med. 2010;122(4):112-28.

20. Katz N, Kopecky EA, O'Connor M, Brown RH, Fleming AB. A phase 3, multicenter, randomized, double-blind, placebo-controlled, safety, tolerability, and efficacy study of Xtampza ER in patients with moderate-to-severe chronic low back pain. Pain. 2015;156(12):2458-67.

21. Kawamata M, Iseki M, Kawakami M, Takahashi M, Kizawa Y, Morita T, et al. Efficacy and safety of controlled-release oxycodone for the management of moderate-to-severe chronic low back pain in Japan: results of an enriched enrollment randomized withdrawal study followed by an open-label extension study. J Pain Res. 2019;12:363-75.

22. Landau CJ, Carr WD, Razzetti AJ, Sessler NE, Munera C, Ripa SR. Buprenorphine transdermal delivery system in adults with persistent noncancer-related pain syndromes who require opioid therapy: a multicenter, 5-week run-in and randomized, double-blind maintenance-of-analgesia study. Clin Ther. 2007;29(10):2179-93.

23. Lynch ME, Clark AJ, Sawynok J. Intravenous adenosine alleviates neuropathic pain: a double blind placebo controlled crossover trial using an enriched enrolment design. Pain. 2003;103(1-2):111-7.

24. Plazzi G, Ruoff C, Lecendreux M, Dauvilliers Y, Rosen CL, Black J, et al. Treatment of paediatric narcolepsy with sodium oxybate: a double-blind, placebo-controlled, randomised-withdrawal multicentre study and open-label investigation. Lancet Child Adolesc Health. 2018;2(7):483-94.

25. Portenoy RK, Messina J, Xie F, Peppin J. Fentanyl buccal tablet (FBT) for relief of breakthrough pain in opioid-treated patients with chronic low back pain: a randomized, placebo-controlled study. Curr Med Res Opin. 2007;23(1):223-33.

26. Raskin P, Huffman C, Toth C, Asmus MJ, Messig M, Sanchez RJ, et al. Pregabalin in patients with inadequately treated painful diabetic peripheral neuropathy: a randomized withdrawal trial. Clin J Pain. 2014;30(5):379-90.

27. Ratain MJ, Eisen T, Stadler WM, Flaherty KT, Kaye SB, Rosner GL, et al. Phase II placebo-controlled randomized discontinuation trial of sorafenib in patients with metastatic renal cell carcinoma. J Clin Oncol. 2006;24(16):2505-12.

28. Rauck RL, Nalamachu S, Wild JE, Walker GS, Robinson CY, Davis CS, et al. Single-entity hydrocodone extended-release capsules in opioid-tolerant subjects with moderate-to-severe chronic low back pain: a randomized double-blind, placebo-controlled study. Pain Med. 2014;15(6):975-85.

29. Rauck RL, Hale ME, Bass A, Bramson C, Pixton G, Wilson JG, et al. A randomized double-blind, placebo-controlled efficacy and safety study of ALO-02 (extended-release oxycodone surrounding sequestered naltrexone) for moderate-to-severe chronic low back pain treatment. Pain. 2015;156(9):1660-9.

30. Schnitzer TJ, Gray WL, Paster RZ, Kamin M. Efficacy of tramadol in treatment of chronic low back pain. J Rheumatol. 2000;27(3):772-8.

31. Schnitzer TJ, Kamin M, Olson WH. Tramadol allows reduction of naproxen dose among patients with naproxen-responsive osteoarthritis pain: a randomized, double-blind, placebo-controlled study. Arthritis Rheum. 1999;42(7):1370-7.

32. Schwartz S, Etropolski M, Shapiro DY, Okamoto A, Lange R, Haeussler J, et al. Safety and efficacy of tapentadol ER in patients with painful diabetic peripheral neuropathy: results of a randomized-withdrawal, placebo-controlled trial. Curr Med Res Opin. 2011;27(1):151-62.

33. Simpson DM, Messina J, Xie F, Hale M. Fentanyl buccal tablet for the relief of breakthrough pain in opioid-tolerant adult patients with chronic neuropathic pain: a multicenter, randomized, double-blind, placebo-controlled study. Clin Ther. 2007;29(4):588-601.

34. Steiner DJ, Sitar S, Wen W, Sawyerr G, Munera C, Ripa SR, et al. Efficacy and safety of the seven-day buprenorphine transdermal system in opioid-naïve patients with moderate to severe chronic low back pain: an enriched, randomized, double-blind, placebo-controlled study. J Pain Symptom Manage. 2011;42(6):903-17.

35. Strollo PJ Jr, Hedner J, Collop N, Lorch DG Jr, Chen D, Carter LP, et al. Solriamfetol for the treatment of excessive sleepiness in OSA: a placebo-controlled randomized withdrawal study. Chest. 2019;155(2):364-74.

36. Tandon R, Cucchiaro J, Phillips D, Hernandez D, Mao Y, Pikalov A, et al. A double-blind, placebo-controlled, randomized withdrawal study of lurasidone for the maintenance of efficacy in patients with schizophrenia. J Psychopharmacol. 2016;30(1):69-77.

37. Vinik AI, Shapiro DY, Rauschkolb C, Lange B, Karcher K, Pennett D, et al. A randomized withdrawal, placebo-controlled study evaluating the efficacy and tolerability of tapentadol extended release in patients with chronic painful diabetic peripheral neuropathy. Diabetes Care. 2014;37(8):2302-9.

38. Vondrackova D, Leyendecker P, Meissner W, Hopp M, Szombati I, Hermanns K, et al. Analgesic efficacy and safety of oxycodone in combination with naloxone as prolonged release tablets in patients with moderate to severe chronic pain. J Pain. 2008;9(12):1144-54.

39. Vorsanger GJ, Xiang J, Gana TJ, Pascual MLG, Fleming RR. Extended-release tramadol (tramadol ER) in the treatment of chronic low back pain. J Opioid Manag. 2008;4(2):87-97.

40. Wen W, Sitar S, Lynch SY, He E, Ripa SR. A multicenter, randomized, double-blind, placebo-controlled trial to assess the efficacy and safety of single-entity, once-daily hydrocodone tablets in patients with uncontrolled moderate to severe chronic low back pain. Expert Opin Pharmacother. 2015;16(11):1593-606.

41. Zakrzewska JM, Palmer J, Morisset V, Giblin GM, Obermann M, Ettlin DA, et al. Safety and efficacy of a Nav1.7 selective sodium channel blocker in patients with trigeminal neuralgia: a double-blind, placebo-controlled, randomized withdrawal phase 2a trial. Lancet Neurol. 2017;16(4):291-300.
